# A comprehensive multi-omics analysis identifies a robust scoring system for cancer-associated fibroblasts and intervention targets in colorectal cancer

**DOI:** 10.1007/s00432-023-05548-7

**Published:** 2024-03-13

**Authors:** Feng Wang, Zhenlin Li, Tianlei Xu, Qian Zhang, Tianyi Ma, Sijia Li, Xiaohui Wang

**Affiliations:** 1 Department of Gastrointestinal Surgery, Beijing Tsinghua Changgung Hospital, School of Clinical Medicine, Tsinghua University, Beijing, China; 2 Department of Surgical Clinical, School of Heze Medical College, Heze, China; 3 Department of General Surgery, Xuanwu Hospital, Capital Medical University, Beijing, China

**Keywords:** CAF, Colorectal cancer, Prognosis, ICB response biomarkers

## Abstract

**Background:**

Cancer-associated fibroblasts (CAF) play a critical role in promoting tumor growth, metastasis, and immune evasion. While numerous studies have investigated CAF, there remains a paucity of research on their clinical application in colorectal cancer (CRC).

**Methods:**

In this study, we collected differentially expressed genes between CAF and normal fibroblasts (NF) from previous CRC studies, and utilized machine learning analysis to differentiate two distinct subtypes of CAF in CRC. To enable practical application, a CAF-related genes (CAFGs) scoring system was developed based on multivariate Cox regression. We then conducted functional enrichment analysis, Kaplan–Meier plot, consensus molecular subtypes (CMS) classification, and Tumor Immune Dysfunction and Exclusion (TIDE) algorithm to investigate the relationship between the CAFGs scoring system and various biological mechanisms, prognostic value, tumor microenvironment, and response to immune checkpoint blockade (ICB) therapy. Moreover, single-cell transcriptomics and proteomics analyses have been employed to validate the significance of scoring system-related molecules in the identity and function of CAF.

**Results:**

We unveiled significant distinctions in tumor immune status and prognosis not only between the CAF clusters, but also across high and low CAFGs groups. Specifically, patients in CAF cluster 2 or with high CAFGs scores exhibited higher CAF markers and were enriched for CAF-related biological pathways such as epithelial–mesenchymal transition (EMT) and angiogenesis. In addition, CAFGs score was identified as a risk index and correlated with poor overall survival (OS), progression-free survival (PFS), disease-free survival (DFS), and recurrence-free survival (RFS). High CAFGs scores were observed in patients with advanced stages, CMS4, as well as lymphatic invasion. Furthermore, elevated CAFG scores in patients signified a suppressive tumor microenvironment characterized by the upregulation of programmed death-ligand 1 (PD-L1), T-cell dysfunction, exclusion, and TIDE score. And high CAFGs scores can differentiate patients with lower response rates and poor prognosis under ICB therapy. Notably, single-cell transcriptomics and proteomics analyses identified several molecules related to CAF identity and function, such as *FSTL1*, *IGFBP7*, and *FBN1*.

**Conclusion:**

We constructed a robust CAFGs score system with clinical significance using multiple CRC cohorts. In addition, we identified several molecules related to CAF identity and function that could be potential intervention targets for CRC patients.

**Supplementary Information:**

The online version contains supplementary material available at 10.1007/s00432-023-05548-7.

## Introduction

According to the 2020 global cancer statistics, colorectal cancer (CRC) is one of the most common malignant tumors in the world, ranking third and second in incidence and mortality, respectively (Sung et al. 2021). At present, there are many treatments for CRC, including radiotherapy and chemotherapy, immunotherapy, surgical treatment, which greatly improve the survival of patients. However, the response of patients to treatment is different, resulting in different outcomes. In view of this phenomenon, CRC consensus molecular subtypes (CMS) (Guinney et al. 2015), a recently established classification based on the transcriptome data may provide an explanation, in which the CMS4 (mesenchymal), characterized by prominent transforming growth factor β (TGF-β) activation, stromal invasion, and angiogenesis, are associated with poor prognosis, and suggest the importance of cancer-associated fibroblasts (CAF), the major component of the stroma, to the prognosis of CRC patients.

Cancer-associated fibroblasts (CAF), one of the plastic cells types in TME, has different origins, including resident fibroblasts, pericytes, endothelial cells, adipocytes, and so on. For the origin of CAF in CRC, most of CAF have been proved to be produced by the proliferation of intestinal pericryptal leptin receptor (Lepr) + cells (Kobayashi et al. 2022). In the progression of CRC, CAF may promote or inhibit tumor, but the prevailing idea is considered to promote tumor. CAF contribute significantly to tumorigenesis (Kasashima et al. 2021; Zhu et al. 2019), angiogenesis (Unterleuthner et al. 2020; Pape et al. 2020), immunosuppression (Li et al. 2019), metastasis, and drug resistance (Hu et al. 2019) in CRC. CAF has several subtypes, among which inflammatory CAF (iCAF) and myofibroblast CAF (myCAF) have been studied. In pancreatic cancer, iCAF can secrete high levels of inflammatory cytokines far away from tumor cells, while myCAF can produce matrix contractile phenotype, and are adjacent to tumor cells (Öhlund et al. 2017). In CRC, myCAF and iCAF are induced by high and low levels of Wnt activity, respectively. And iCAF promote EMT phenotype, while myCAF reverse the phenotype (Mosa et al. 2020). In preoperative radiotherapy and chemotherapy for rectal cancer, the inflammatory polarization of CAF leads to the resistance of radiotherapy and chemotherapy, and promotes tumor progression (Nicolas et al. 2022). After cetuximab treatment, CAF can make neighboring cancer cells resistant to cetuximab in CRC (Garvey et al. 2020). In addition, it has been reported that some molecules expressing on CAF, including *WNT2* (Huang et al. 2022), *WNT5a* (Hirashima et al. 2021), *CLEC3B* (Zhu et al. 2019), *IFNAR1* (Cho et al. 2020), IL-34 (Franzè et al. 2020), miR-1246 (Si et al. 2021), and *FAP* (Yuan et al. 2021), play an important role in the development of CRC, and are expected to become a target for anticancer therapy. And many models have been established to study CAF in CRC, such as 3D model of tumor tissue in vitro to simulate the physiological function of cells in vivo (Chen et al. 2020), in vitro co-culture model of patient-derived organ-like organ (PDO) and patient-derived CAF (Luo et al. 2021; Naruse et al. 2021), and mouse xenotransplantation model co-injected with CAF and CRC cell line (Fernando-Macías et al. 2020). These models are helpful to investigate the functions of various subtypes of CAF, and find the therapy strategies targeting CAF in CRC. Although there have been many in vitro and in vivo experiments focusing on the functional characteristics of CAF, applying the research findings in clinical practice is still an urgent issue.

Previous studies have shown that the heterogeneity of CAF significantly correlates with the efficacy of ICB therapy. For example, Wang et al. have used single-cell RNA-seq to analyze the heterogeneity of CAF, and identified a novel fibroblast subtype, independent of iCAF and myCAF, which was termed meCAF characterized by highly active glycolysis, and associated with better response to anti-PD-1 therapy in pancreatic ductal adenocarcinoma (Wang et al. 2021). In addition, Kalluri's laboratory identified tumor-restraining cancer-associated fibroblasts (rCAFs) that enhance the effectiveness of immune checkpoint inhibitors (Chen et al. 2021). Researchers from Jørgensen's team clearly demonstrated some specific CAF lineage supports anti-tumor immunity (Hutton et al. 2021). Also, an interesting rCAFs subset has been reported from a Japanese group (Miyai et al. 2022; Ando et al. 2022), that clearly associates with favorable response to immune checkpoint inhibitors. However, compared with bulk RNA-seq, single-cell RNA-seq is dramatically expensive, and not suitable for wide clinical application.

In this study, we performed unsupervised clustering in a large-scale CRC cohort based on CAF-related genes, and identified two groups of patients with distinct biological functions related to CAF (named CAF cluster 1 and 2). Furthermore, we constructed a novel CAF scoring system composed of 15 genes, which were associated with poor overall, disease-free, recurrence-free, and disease-specific survival (OS, DFS, RFS and DSS), and had the potential to guide ICB treatment. Moreover, single-cell sequencing and proteomics data suggest that these 15 genes might be linked to CAF identity and function, thus rendering them potential therapeutic targets for CAF intervention in CRC.

## Results

### Construction of CAF clusters with different prognosis and immune states

**Fig. 1:**
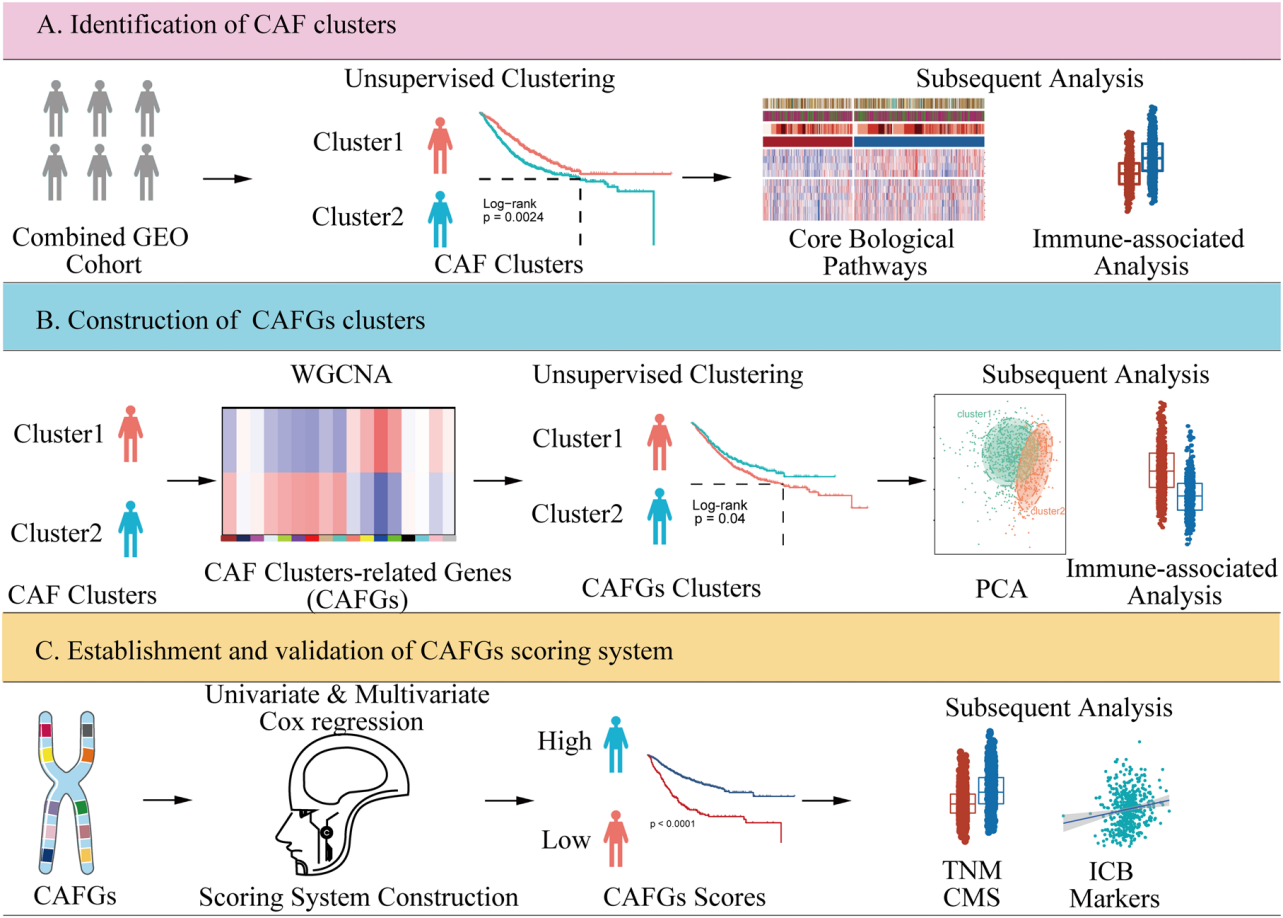
Flow diagram of this study

**Fig. 2:**
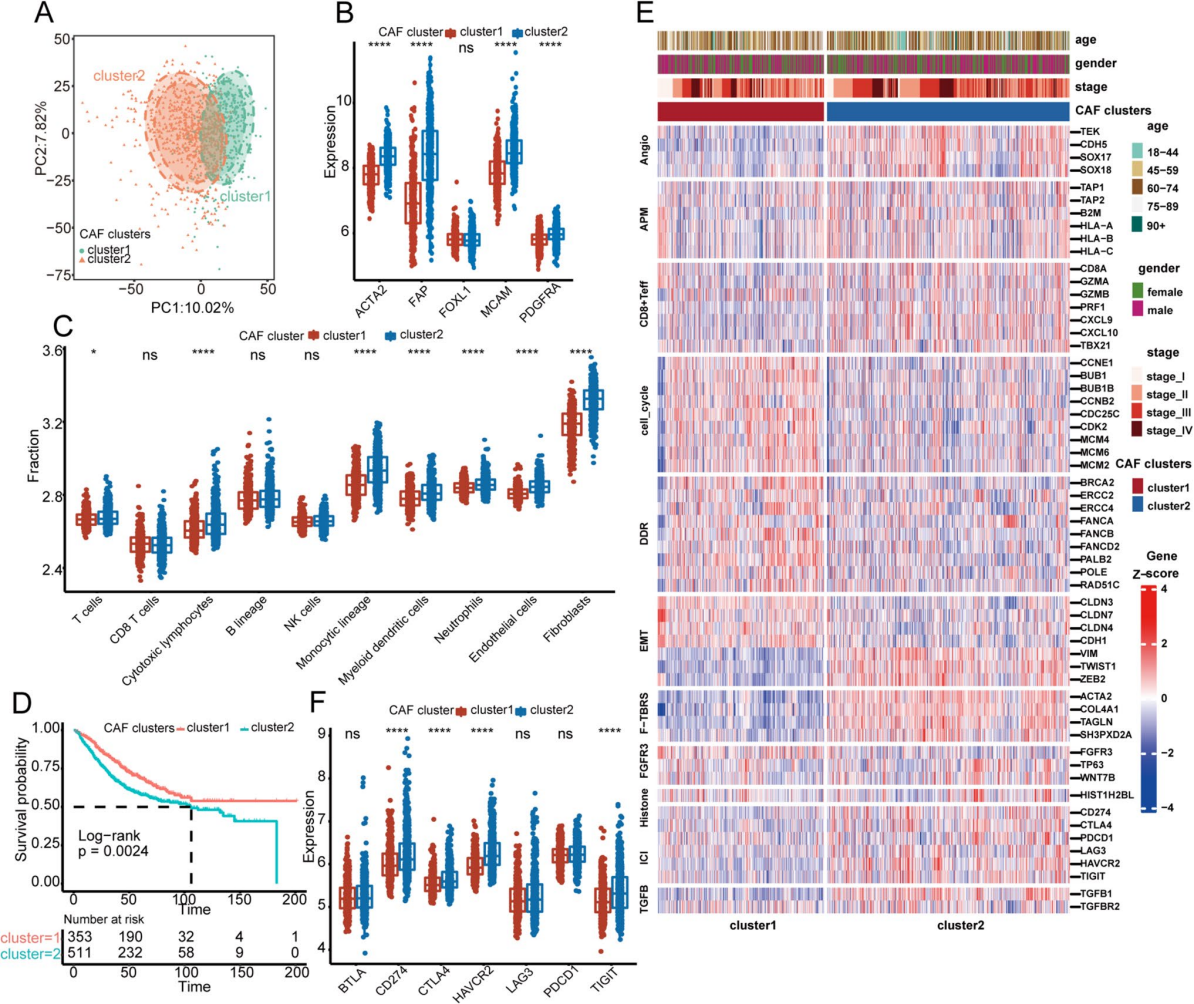
Clinical outcomes and biological functions between ERS clusters. **A** The different gene expression patterns between CAF clusters 1 and 2 showed by PCA plot. Each point represents one sample. **B** The boxplot reveals the expression levels of CAF markers between CAF clusters. **C** The fraction of immune cells infiltrating in TME between CAF clusters inferred by *MCPcounter*. **D** KM plot shows the overall survival between CAF clusters 1 and 2 in the GEO combined cohort. The log-rank test was used in the survival analysis. **E** The heatmap reveals the relationships between CAF clusters and 11 critical biological pathways. Rows of the heat map represent gene expression grouped by pathway. Red and blue colors represent high and low expression, respectively. **F** The mRNA expression levels of several common inhibitory immune checkpoints between the CAF clusters. *, **, ***, and **** represent a* p* value less than 0.05, 0.01, 0.001, and 0.0001, respectively. The difference between the two groups was tested using the Wilcox test

The study flow diagram is presented in Fig. 1. First, we collected 596 differentially expressed genes (named CAF genes) between CAF and normal fibroblasts (NF) in CRC from a previous study (Herrera et al. 2021) (Supplementary Data 1). To find prognostic genes, we then performed univariate Cox regression analysis in GEO combined cohort, and recognized 115 potential prognostic genes among above genes (Supplementary Data 2). Furthermore, we conducted unsupervised clustering in GEO combined cohort based on 115 prognostic CAF genes using the *ConsesusClusterPlus* R package. As shown in Supplementary Fig. 1, the clustering results were most stable when patients were divided into two groups (defined as CAF cluster 1 and 2). The PCA plot shows significant differences in gene expression profiles between the two clusters (Fig. 2A). Remarkably, we found several previously identified CAF-related markers (Han et al. 2020; Gascard and Tlsty 2016), including *ACTA2*, *FAP*, *FOXL1*, *MCAM*, and *PDGFRA*, were substantially upregulated in CAF cluster 2 relative to CAF cluster 1 (Fig. 2B), suggesting that the distinct status of CAF is correlated to group classification. In addition, *MCPcounter* analysis showed that fibroblast scores of patients in CAF cluster 2 were markedly higher than patients in CAF cluster 1 (Fig. 2C), while KM survival analysis illustrated that the OS was notably better for patients in CAF cluster 1 than those in CAF cluster 2 (Fig. 2D, log-rank test, *p* = 0.0024). These results imply that the varied CAF status significantly impacts the survival of patients with CRC.

To dissect the underlying biological functions between the CAF clusters, we collected 11 tumorigenesis-related pathways from prior research. Our findings showed that the angiogenesis, TGF β, and F-TBRS signature scores were dramatically elevated in CAF cluster 2 compared to CAF cluster 1 (Fig. 2E). In addition, the gene expression of APM, CD8 + Teff, and ICI pathways is dramatically increased in CAF cluster 2 (Fig. 2E, F), suggesting potentially distinct biological functions between the two groups. To further understand the immune status between the two groups, we analyzed the expression patterns of 122 immunomodulators (including *MHC*, receptors, chemokines, and immunostimulants) between CAF cluster 1 and 2, most of which were highly expressed in CAF cluster 2 (Supplementary Fig. 2A). The abundance of most tumor infiltrating lymphocytes inferred by *MCPcounter* analysis was also significantly higher in CAF cluster 2 than in CAF cluster 1, such as T cells, cytotoxic lymphocytes, and neutrophils (Fig. 2C). Besides, ssGSEA-inferred adaptive and innate immunity scores were also significantly increased in CAF cluster 2 (Supplementary Fig. 2B), whereas the expression of immune checkpoint molecules was significantly higher in CAF cluster 2 than in CAF cluster 1. These results demonstrate that the CAF clusters exhibit distinct immune microenvironments, with CAF cluster 2 exhibiting an inhibitory immune microenvironment.

### Identification of the key genes affecting the prognosis of patients in different CAF clusters

**Fig. 3:**
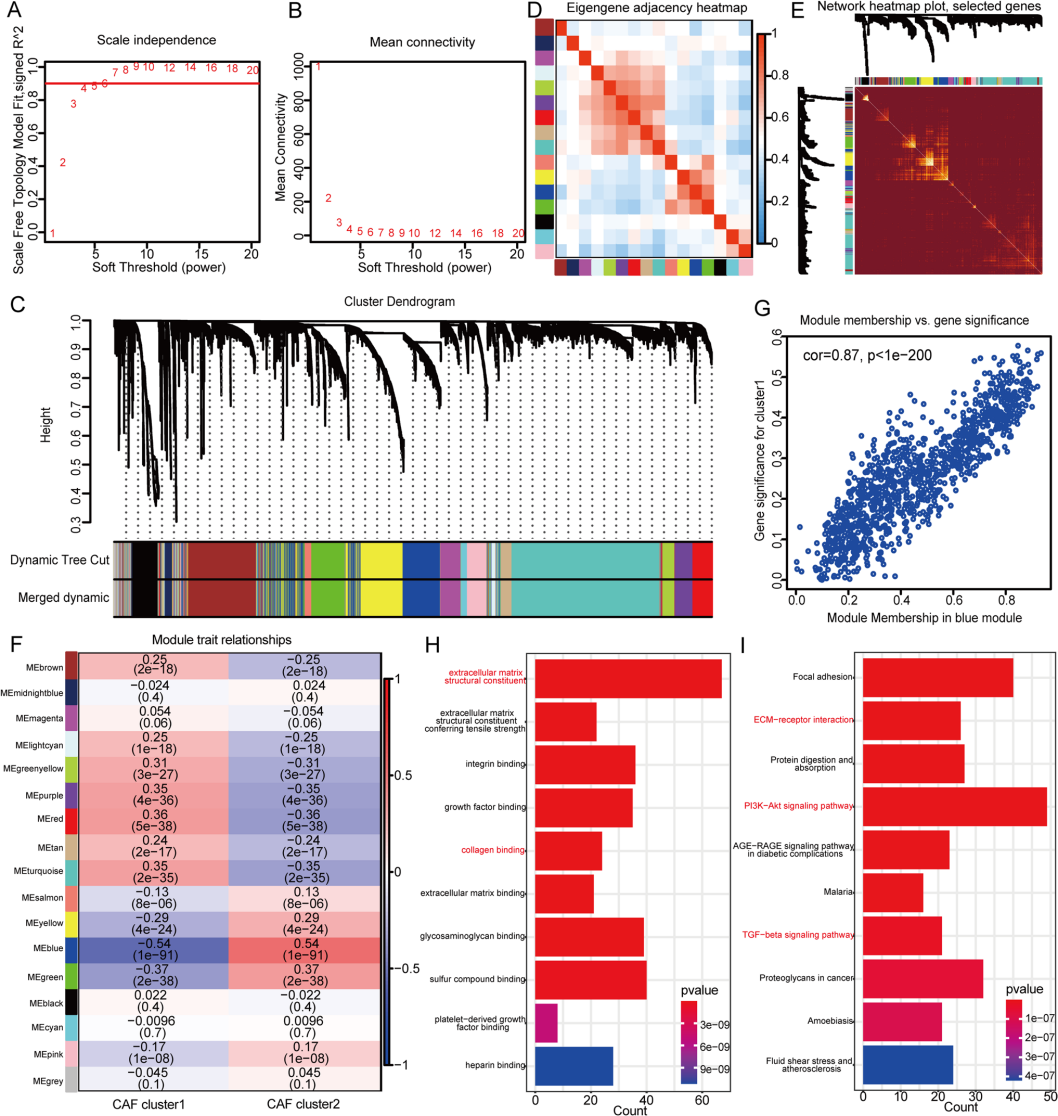
WGCNA analysis identifies the key CAF-related genes. **A**, **B** The plots show scale-free fit index and the mean connectivity in various soft-thresholding power values, identifying soft-thresholding power value = 4 in the next analysis. **C** Hierarchical clustering dendrogram of co-expressed genes in modules. **D**, **E** The heatmaps display the correlations between different modules. **F** Module trait relationships show the correlation between module eigengenes and CAF clusters. Each row contains the corresponding correlation value and *p* value. Red and blue colors represent the positive and negative correlations, respectively. **G** The scatter plot reveals the significant correlation between module eigengenes and CAF cluster 1 in blue modules (cor = 0.87, *p* < 1e-200). **H**, **I** The barplots show the top ten GO and KEGG enrichment terms in blue module. The enriched pathways mentioned in our paper are highlighted as red words. An adjusted *p* value < 0.05 is considered as statistically significant

**Fig. 4:**
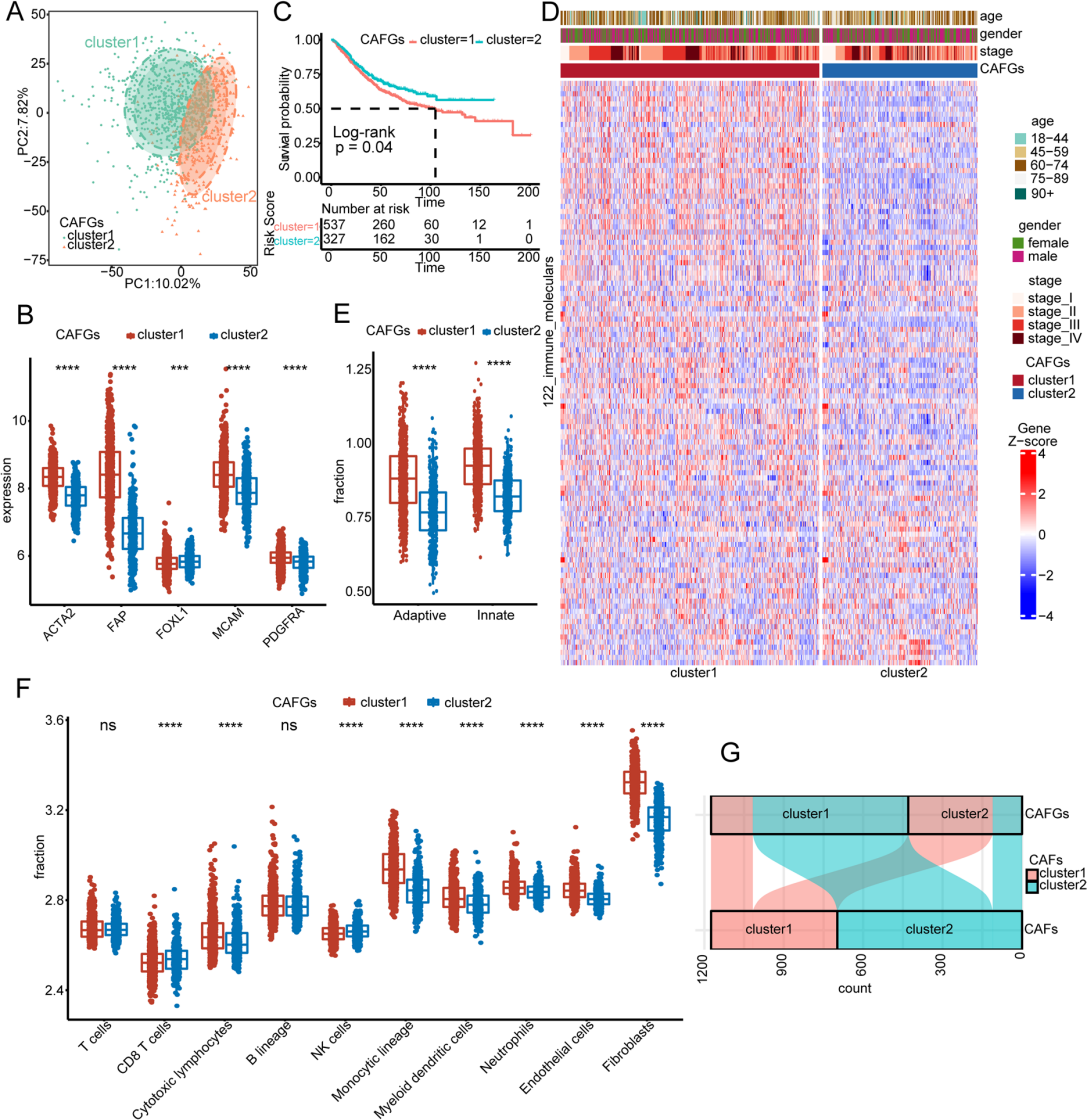
Clinical outcomes and biological functions between CAFGs clusters. **A** The PCA plot shows the different gene expression patterns between CAFGs clusters. **B** The expression levels of CAF markers between CAFGs clusters. **C** KM plot shows the OS of CAFGs clusters in the GEO combined cohort. The log-rank test was used for KM survival analysis. **D** The mRNA expressions of 122 immunomodulators between the CAFGs clusters. **E** The enrichment scores of adaptive and innate immunity inferred by ssGSEA analysis between CAFGs clusters. **F** The distribution of immune cells infiltrating in the TME inferred by MCP-counter algorithm between CAFGs clusters. **G** The Sankey plot revels the relationships between the CAF and CAFGs clusters. *, **, ***, and **** represent a *p* value less than 0.05, 0.01, 0.001, and 0.0001, respectively. The difference between the two groups was assessed using the Wilcox test

To identify the key genes affecting the survival of patients in CAF clusters, WGCNA analysis was carried out, and CAF clusters 1 and 2 were used as the traits. As shown in Fig. 3A, B, the soft threshold power of β was set as 4 when scale-free topology model-fit *R* = 0.9. Then we identified 16 modules, except the grey module (Fig. 3C–E). Module-trait heatmap shows that the blue module was the most closely related to CAF clusters (Fig. 3F, G). And the biological functions of genes in the blue modules were explored using GO and KEGG analysis. When the adjusted p value was less than 0.05, 27 and 12 items were identified by GO and KEGG analyses, respectively (Supplementary Data 3, 4). The top ten enrichment items in GO and KEGG analyses included extracellular matrix structural constituent, collagen binding, ECM–receptor interaction, PI3K-Akt, and TGF-beta signaling pathway (Fig. 3H, I), which were in consistent with the functions of CAF. Therefore, the blue module containing 1089 genes was identified as the key module, among which 100 genes meeting GS > 0.2 and MM > 0.8 were considered as the critical genes related to CAF clusters (CAFGs). In addition, univariate cox regression analysis identified 43 of the 100 CAFGs was prognostic genes with a p value less than 0.01. Then these genes were again used for unsupervised clustering in GEO combined cohort (Supplementary Data 5). The detail processes of unsupervised clustering are shown in the Supplementary Fig. 3. Surprisingly, when the patients were again divided into two groups, defined as CAFGs clusters 1 and 2, the clustering results were the most stable. The PCA plot shows that there were significant differences in gene expression profiles between the CAFGs clusters (Fig. 4A). The markers associated with CAF and the fibroblast score inferred by *MCPcounter* analysis were significantly higher in CAFGs cluster 1 than in CAFGs cluster 2 (Fig. 4B, F). Consistently, the OS of CAFGs cluster 1 was significantly worse than that of CAFGs cluster 2 (Fig. 4C, log-rank test, p 0.04). In addition, the heatmap of 11 tumorigenesis-related pathways shows that the angiogenesis, TGF β, and F-TBRS signature score of CAFGs cluster 1 were higher than that of CAFGs cluster 2, and the levels of APM, CD8 + Teff, and ICI signatures were also increased in CAFGs cluster 1 (Supplementary Fig. 4A, B). Besides, the expression of 122 immunomodulators (Fig. 4D), adaptive and innate immunity (Fig. 4E), and most tumor infiltrating lymphocytes (Fig. 4F, Supplementary Fig. 4C) were higher in CAFGs cluster 1 than in CAFGs cluster 2. Furthermore, we observed most of the patients consisting of CAFGs cluster 1 were from CAF cluster 2 (Fig. 4G). Therefore, these findings demonstrated the crucial roles of CAFGs, which can reproduce the biological category of CAF clusters.

### Colorectal cancer patients with high CAFGs score have poor outcomes in multiple colorectal cohorts

**Fig. 5:**
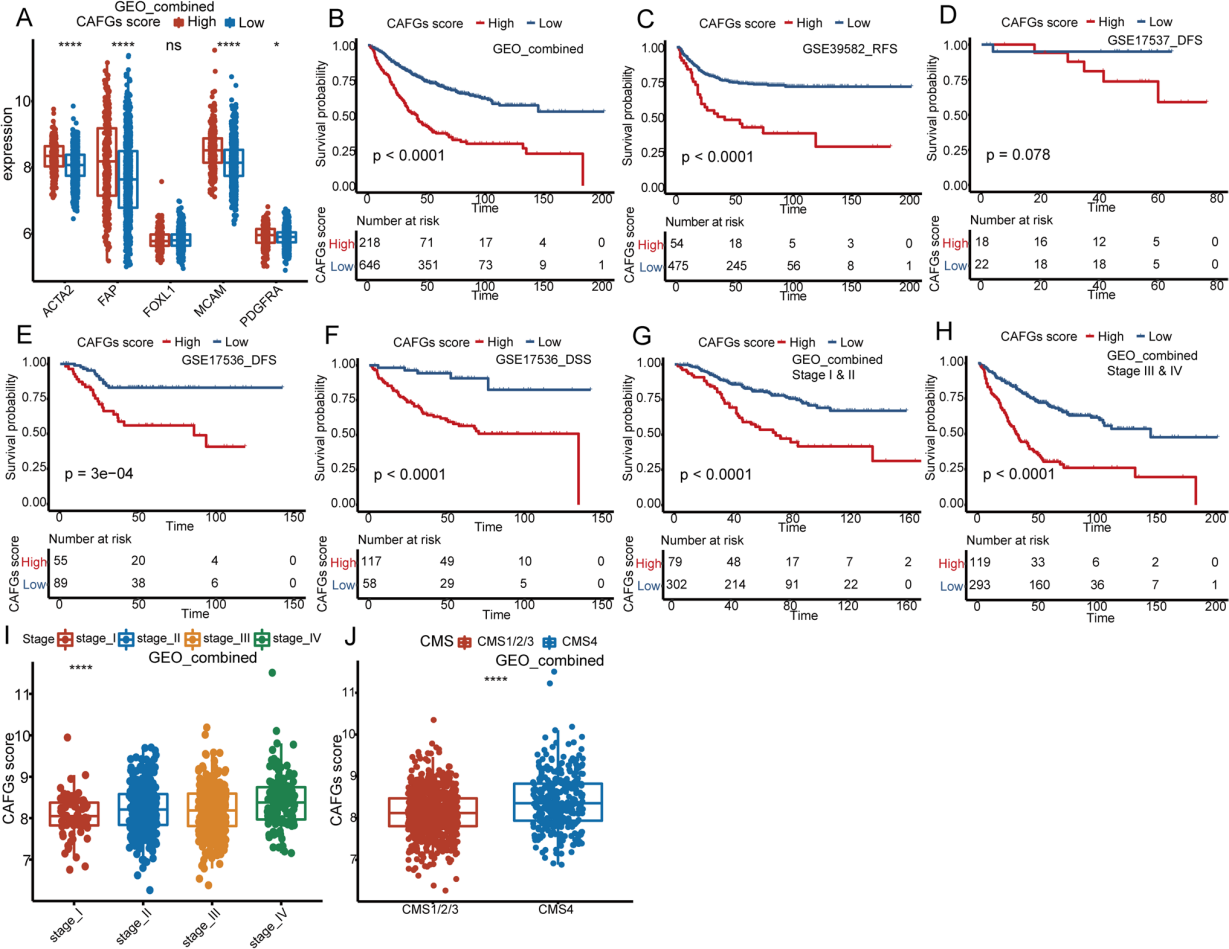
Clinical significance of CAFGs scoring system in GEO combined cohort. **A** The boxplot reveals the expression levels of CAF markers between patients with high and low CAFGs scores. **B** The OS analysis of CAFGs scores in the GEO combined cohort. **C**–**F** KM plots show the influence of CAFGs scores on RFS, DFS, and DSS in GSE39583, GSE17537, and GSE17536 cohorts. **G**–**H** The OS analysis of CAFGs scores in early (stage I and II) and advanced stages (stage III and IV) in the GEO combined cohort. The log-rank test was used in the survival analysis. **I** The distribution of CAFGs scores in different TNM stages. The statistic differences are assessed by the Kruskal test. The log-rank test was used for KM survival analysis. **J** The distribution of CAFGs scores between CMS1-3 and CMS4. The statistic differences are assessed by the Wilcox test. *, **, ***, and **** represent a *p* value less than 0.05, 0.01, 0.001, and 0.0001, respectively

**Fig. 6:**
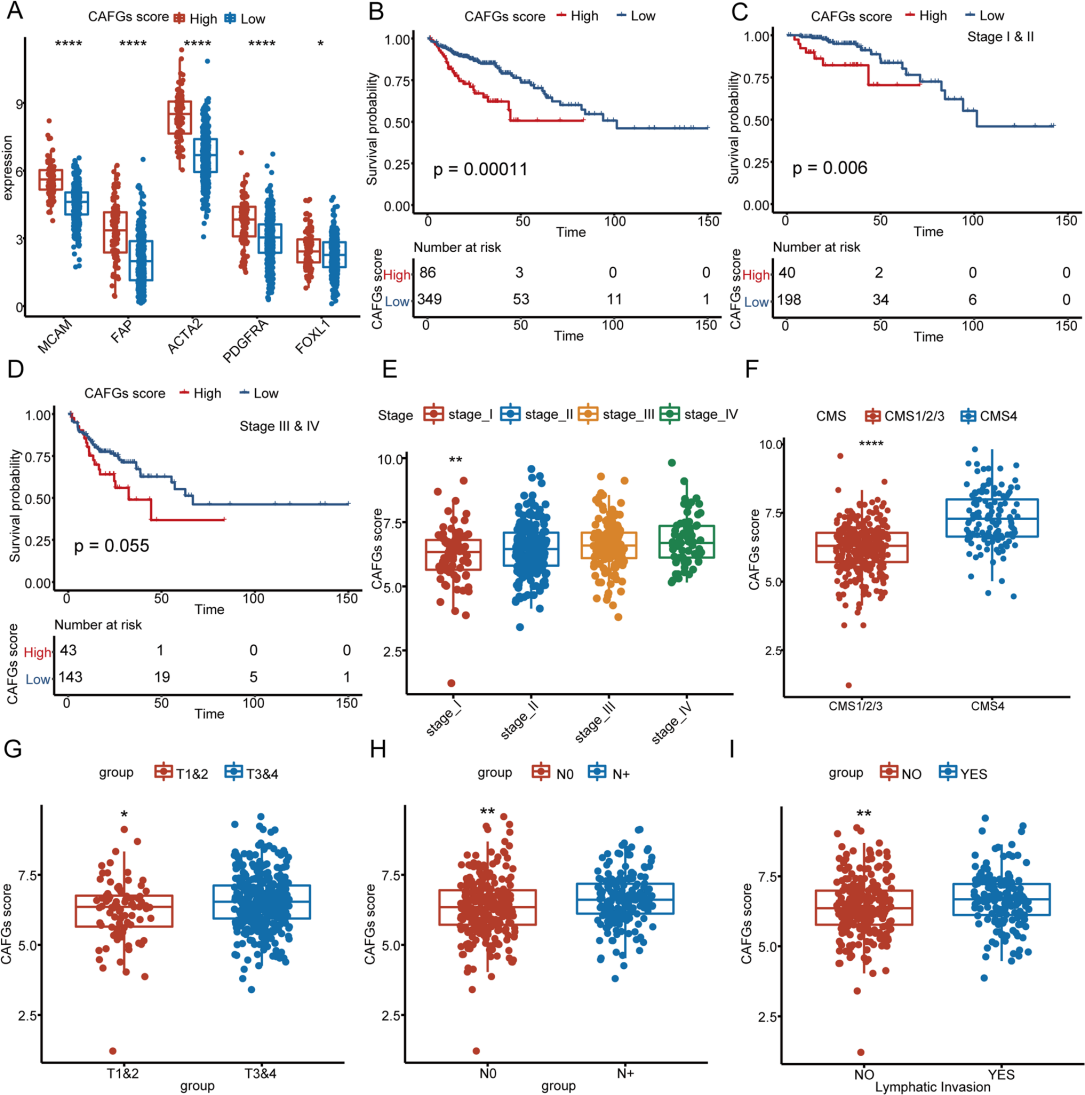
Validation of the clinical significance of CAFGs scoring system in TCGA COAD cohort. **A** The expression levels of CAF markers between patients with high and low CAFGs scores in TCGA COAD cohort. **B** The OS analysis of CAFGs scores in the TCGA COAD cohort. **C**–**D** The OS analysis of CAFGs scores in early and advanced stages in the TCGA COAD cohort. The log-rank test was used in the survival analysis. **E** The distribution of CAFGs scores in different TNM stages. The statistic differences are assessed by the Kruskal test. **F**–**I** The boxplot shows the CAFGs scores in different groups of CMS classification, *T* stage, *N* stage, and lymphatic invasion. The statistic differences are assessed by the Wilcox test. *, **, ***, and **** represent a *p* value less than 0.05, 0.01, 0.001, and 0.0001, respectively

Gene model plays an important role in clinical application. To construct a scoring model for clinical application, the CAFGs meeting a *p* value < 0.2 in univariate analysis were included in multivariate unicox regression analysis. Finally, 15 genes with a *p* value < 0.05 were obtained, including *FNDC1*, *FRMD6*, *FBN1*, *RAB31*, *GLT8D2*, *COL1A2*, *GLIS2*, *COL8A1*, *GPC6*, *COL3A1*, *PRICKLE1*, *FSTL1*, *HLX*, *IGFBP7*, and *EFS*. These genes were considered as the important prognostic factors, and again incorporated in the cox model. Next, using the expression values of these 15 genes and their corresponding regression coefficients, a scoring model, named CAFGs scoring system, was constructed (Supplementary Data 6). We then included TCGA COAD and GSE39582 cohorts as external and internal validation sets. According to the best cutoff value determined by *survminer* R package, patients in these cohort were divided into high and low CAFGs score groups (Supplementary Data 7). We found that the expression levels of CAF markers were significantly higher in the high CAFGs score group than in the low CAFGs score group (Fig. 5A: GEO combined; Fig. 6A: TCGA COAD; Supplementary Fig. 5A: GSE39582). Then we observed that patients with high CAFGs scores showed worse OS in GEO combined cohort, which were also verified in the internal and external cohorts (Fig. 5B: GEO-combined; Fig. 6B: TCGA COAD cohort; Supplementary Fig. 5B: GSE39582). In additional, we analyzed the DFS, RFS, and DSS in GSE39582, GSE17536, and GSE17537 cohorts. The results show that the RFS, DFS, and DSS of patients with high CAFGs scores were also significantly worse than that of patients with low CAFGs scores (Fig. 5C–F, GSE39582 RFS, GSE17537 DFS, GSE17536 DFS, GSE17536 DSS). Further analysis demonstrated that high CAFGs scores also represented poor OS in both early (stage I and II) and advanced (stage III and IV) patients (Figs. 5G, H; 6C, D; Supplementary Fig. 5C, D). And the CAFGs scores of patients in stage III and IV were significantly higher than that of patients in stage I and II (Figs. 5I; 6E; Supplementary Fig. 5E). CMS classification, a widely used classification system in CRC, has strong prognostic implications, and includes four subtypes, such as CMS1 (MSI immune), CMS2 (canonical), CMS3 (metabolic), and CMS4 (mesenchymal). To our surprise, CAFGs scores of patients in CMS subtype 4 were significantly higher than those of patients in CMS subtypes 1–3 in the combined GEO cohort, as well as TCGA COAD and GSE39582 cohorts (Figs. 5J; 6F; Supplementary Fig. 5F; Supplementary Data 8). CMS subtype 4 is mesenchymal subtype characterized by prominent transforming growth factor β (TGF-β) activation, stromal invasion, and angiogenesis; and has been reported to be associated with poor prognosis.

Next, we conducted a comprehensive investigation of the CAFGs scoring system in the TCGA COAD cohort and examined its association with various clinicopathologic features, such as T, N, and M stages, as well as venous and lymphatic invasion. Our observations revealed that patients in the T3 and 4 stage, N + stage, and those with lymphatic invasion had significantly higher CAFGs scores than patients in the T1and 2 stage, N0 stage, and without lymphatic invasion (Fig. 6G–I; Supplementary Fig. 6). This may underscore the importance of early detection and monitoring of lymphatic invasion and metastasis in patient with high CAFGs score. Moreover, we also performed GSVA using hallmark gene sets from the MSigDB website, and revealed that several signaling pathways, including EMT, were significantly upregulated in patients with high CAFG scores (Supplementary Fig. 7A).

### Patients with a high CAF score develop resistance to immunotherapy

**Fig. 7:**
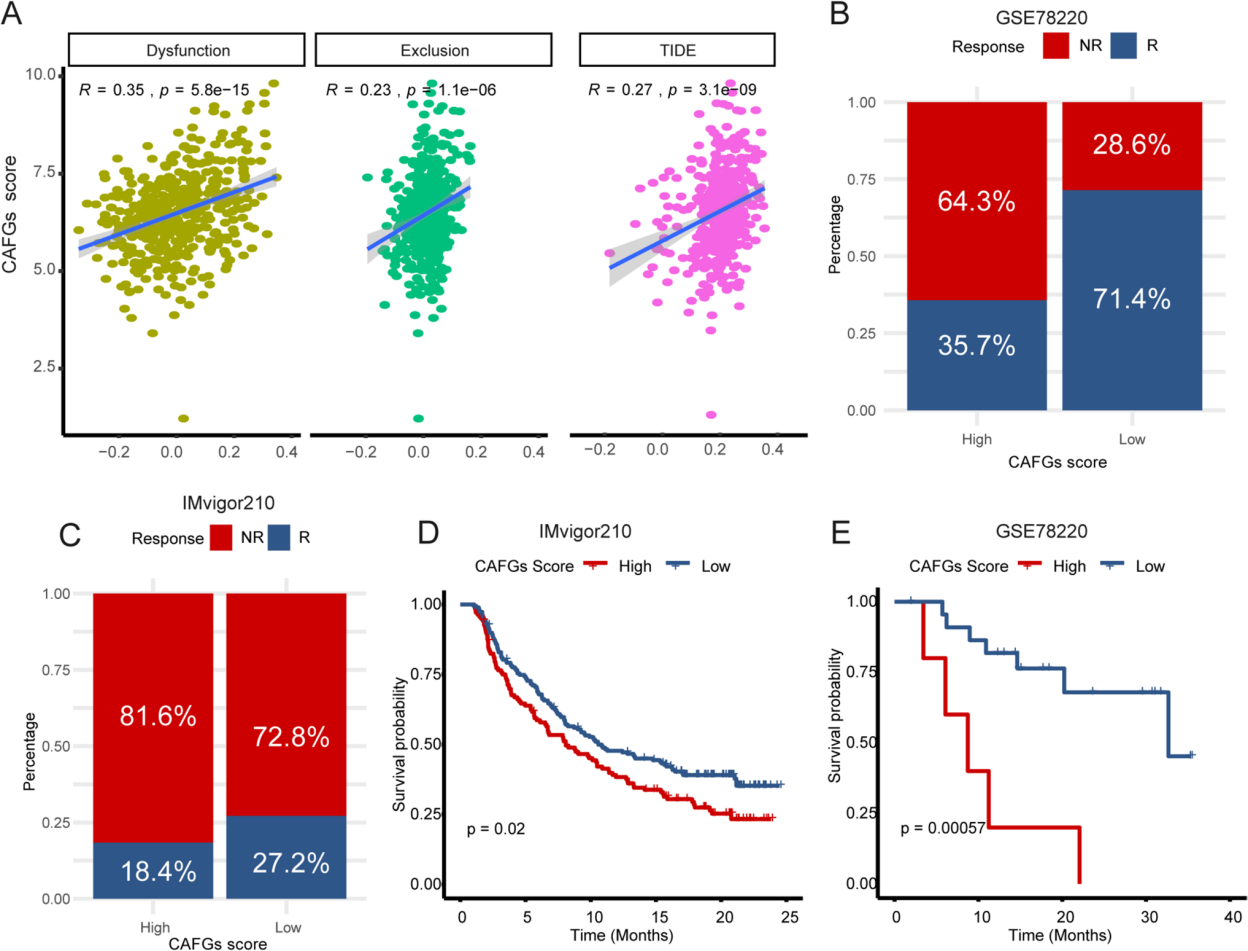
Relationships between CAFGs score and response to ICB therapy. **A** The Pearson correlation analysis between CAFGs scores and T-cell dysfunction (dysfunction), T-cell exclusion (exclusion), and TIDE score. **B**, **C** The stacked histogram shows the distribution of ICB therapy responders and non-responders in the low and high CAFG score groups. **C**, **D** KM plot presents the OS differences of patients with high and low CAFGs scores after receiving ICB therapy. The log-rank test was used for KM survival analysis. *, **, ***, and **** represent a *p* value less than 0.05, 0.01, 0.001, and 0.0001, respectively

It is well known that the therapeutic effect of ICB treatment is closely related to the tumor immune microenvironment, including the abundance of CAF. Therefore, we used TIDE analysis to explore the relationship between CAFGs score and therapeutic response to ICB, which focused on two mechanisms of tumor immune evasion, namely, T-cell dysfunction and exclusion. Through TIDE analysis, we found a significant correlation between CAFGs score and T-cell disfunction score, T-cell exclusion score, and TIDE score (Fig. 7A). In addition, the expression levels of ICI genes were markedly higher in the high CAFG score group compared to the low CAFG score group (Supplementary Fig. 7B). These findings indicated that patients with high CAFGs score may suffer from immune evasion and resistance to immunotherapy. Furthermore, we collected two patient cohorts receiving anti-PD-1 or anti-PD-L1 therapy (GSE78220, and IMvigor210) to analyze the relationship between CAFGs score and ICB efficacy. Indeed, in the high CAFGs score group, we found a higher proportion of no responders (Fig. 7B, C). Consistently, patients with high CAFGs score had significantly worse overall survival (OS) after receiving ICB therapy (Fig. 7D, E). These findings indicated that CAFGs score may have the potential to identify CRC patients who were sensitive to ICB therapy.

### Exploring potential molecules associated with the identity and function of CAF

**Fig. 8:**
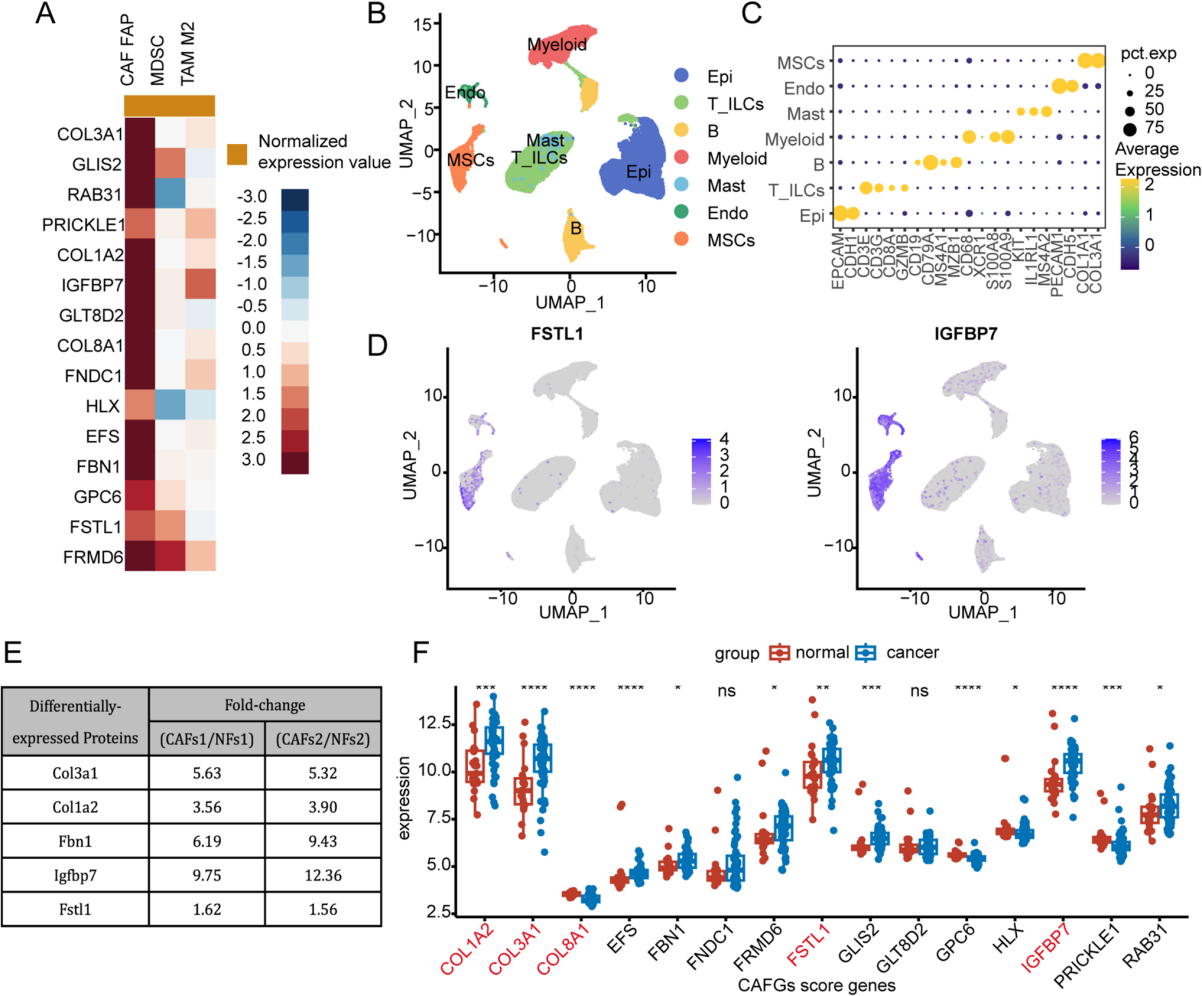
Exploring potential molecules associated with the identity and function of CAF. **A** The correlation between the 15 model genes and the levels of 3 immunosuppressive cell types that drive T-cell exclusion on TIDE website. **B** UMAP plots of cells from 23 primary colorectal cancer samples and 10 matched normal mucosa samples, showing 7 clusters in each plot (data from GSE132465). Each cluster was shown in different colors, including epithelial cells (Epi), T or innate lymphoid cells (T_ILCs), B cells (**B**), myeloid cells (Myeloid), endothelial cells (Endo), mesenchymal stromal cells (MSCs). MSCs can be further categorized into the normal fibroblasts, myofibroblasts, and various other cell types within the stroma. **C** Dot plots showing average expression of known markers in indicated cell clusters. The dot size represents percent of cells expressing the genes in each cluster. **D** Expression levels of selected model genes across different cell clusters illustrated in UMAP plots. **E** The differentially expressed proteins between CAF derived from AOM/DSS-induced CRC mice and normal mice-derived NF. **F** The transcriptional expression levels of 15 model genes between normal and tumor tissues in CRC (data from GSE21510). *, **, ***, and **** represent a *p* value less than 0.05, 0.01, 0.001, and 0.0001, respectively

Several of these 15 genes belonging to CAFGs scoring system were previously reported to be specifically associated with the identity and functionality of CAF, including *COL1A2*, *COL3A1*, *COL8A1*, and *FBN1*, implying the remaining genes may also correlate with CAF. Further analysis of the relationship between these 15 genes and CAF levels in TIDE website revealed that almost all of the genes were significantly correlated with CAF levels (Fig. 8A). Single-cell transcriptomic data from patients with CRC also indicated that nearly all of the genes were significantly expressed in stromal cell types, such as *IGFBP7*, *GLT8D2*, *FSTL1*, *GPC6*, and *FRMD6* (Fig. 8B–D, Supplementary Fig. 8). The proteomic data obtained from CAF derived from AOM/DSS-induced CRC mice and normal mice-derived NF indicate that in addition to commonly known CAF-related proteins (such as *COL3A1*, *COL1A2*, and *FBN1*), *IGFBP7* and *FSTL1* were significantly upregulated in the CAF conditioned medium (Fig. 8E). And transcriptional data reveal that compared to the adjacent non-tumorous samples, expression of *COL3A1*, *COL1A2*, *FBN1*, *IGFBP7*, and *FSTL1* was significantly elevated in the tumor tissue (Fig. 8F). These results suggest that the 15 marker models we identified might play crucial roles in CAF-mediated CRC progression.

## Discussion

Cancer-associated fibroblasts (CAF), an important component of the tumor microenvironment, are involved in tumor initiation, progression, and metastasis (Kalluri 2016; Öhlund et al. 2014; Sahai et al. 2020). Although many promising achievements have been made in the basic research of CAF, applying the research findings in clinical practice is an urgent issue.

In our study, we have established two types of classification based on gene expression related to CAF, named CAF clusters and CAFGs scoring system. Of note, the levels of CAF-related biological terms, such as TGF-b, F-TBRS, angiogenesis, were highly expressed in CAF cluster 2 and high CAFGs score group. Among these groups, CAF markers collected from previous researches significantly increased, including ACTA2, FAP, MCAM, and PDGFRA (Han et al. 2020; Togo et al. 2013). These results indicated that there were more CAF infiltrating in CAF cluster 2 and high CAFGs score group. And the high CAF scoring not only correlated with poor OS, but also with poor DFS, PFS, and DSS. In addition, patients in the CMS4 group had the highest CAFGs scores. CMS is a widely used CRC classification, among which CMS4 is characterized by prominent TGF-β activation, stromal invasion, and angiogenesis, and associated with prognosis, indicating that the CAFGs scoring system may have important application potential.

It encourages us to explore the association between CAF scoring and ICB therapy efficacy. Notably, in our study, patients in CAF cluster 2 and high CAFGs score group also had higher expression of inhibitory immune checkpoints (such as, CD274 [PD-L1], CTLA4, and TIGIT), suggesting that the immune system is in a suppressed state. Also, we found a positive correlation between CAFGs score and T-cell dysfunction, T-cell exclusion, and TIDE scores. Strikingly, there was a higher proportion of non-responders within the high CAFGs score group, and patients exhibiting high CAFGs score had a significantly reduced overall survival (OS) rate following their receipt of ICB therapy.

Moreover, single-cell transcriptomics and proteomics analyses have been employed to validate the significance of scoring-system-related molecules in the identity and function of CAF, including *FNDC1*, *FRMD6*, *FBN1*, *RAB31*, *GLT8D2*, *COL1A2*, *GLIS2*, *COL8A1*, *GPC6*, *COL3A1*, *PRICKLE1*, *FSTL1*, *HLX*, *IGFBP7*, and *EFS*. Consistent with the proteomic data, a previous study established that *FSTL1*, secreted by activated fibroblasts, promotes hepatocellular carcinoma metastasis and stemness (Loh et al. 2021). CAF expressing *IGFBP7* induce colony formation when co-culturing with CRC cells through paracrine tumor–stromal interaction (Rupp et al. 2015). These results suggest that the 15 marker models we identified might play crucial roles in CAF-mediated CRC progression.

Overall, these findings signify the critical role that CAFs play in tumor immune phenotypes and response to ICB therapy, thereby evidencing the potential value of assessing CAFGs as a prognostic tool for those undergoing cancer immunotherapy.

## Materials and methods

### Data source and process

The combined GEO cohort (1175 samples) used in this study was integrated by GSE39582, GSE14333, GSE17536, GSE17537, and GSE72968 cohorts. The transcriptome data of above five cohorts were the microarray data from GPL570 platform. The method of merging multiple data sets and the procedure of removing batch effects were carried out as reported in our previous study (Wang et al. 2022a).The transcriptome data (FPKM) and clinical information of the TCGA COAD cohort were downloaded from the UCSC website (Navarro Gonzalez et al. 2021). Then FPKM was converted to transcripts per kilobase million (TPM) and further log-2 transformed in next analysis.

### Machine learning

The R package *ConsensusClusterPlus* was applied for clustering the combined GEO cohort based on the input genes (Wilkerson and Hayes 2010). To make the clustering result robust, we set the following parameters: 80% item resampling (pItem), 100% gene resampling (pFeature), a maximum evaluated k of 9 (maxK), 1000 resamplings (reps), and pam clustering algorithm (clusterAlg) upon spearman distances (distance).

### Evaluation of immunological characteristics

R package *MCPcounter* (Becht et al. 2016) was applied to infer the abundance of immune cells infiltrating in the TME using the transcriptome data. In addition, the adaptive and innate immune scores of patients were also calculated though *ssGSEA* algorithm in GSVA package, and the parameters were set as follows: method = 'ssgsea', KCDF = 'Gaussian'. And 122 immunomodulators (Supplementary Data 9), including major histocompatibility complex (MHC), receptors, chemokines, and immunostimulants, and several common immune checkpoints with therapeutic potential were collected from previous studies (Charoentong et al. 2017; Auslander et al. 2018; Wang et al. 2022b).

### Weighted correlation network analysis (WGCNA)

Weighted correlation network analysis (WGCNA) enables to identify gene modules most associated with traits (Langfelder and Horvath 2008). In this study, the CAF clusters 1 and 2 were used as the traits. An appropriate soft threshold *β* (*β* = 4 in this study) was used to meet the criteria for the scale-free network. In next steps, WGCNA analysis was performed with default parameters.

### Functional enrichment analyses

Gene Ontology (GO) and Kyoto Encyclopedia of Genes and Genomes (KEGG) (Kanehisa and Goto 2000) analyses were applied to explore the biological functions of the blue modules in WGCNA using the R package “*clusterprofiler*” (Yu et al. 2012). In addition, GSEA and GSVA analyses were performed using the Hallmark gene sets from MSigDB website with default parameters. An adjusted p value of less than 0.05 was regarded as a statistically significant difference.

### Construction and validation of CAFGs scoring system

First, 100 CAFGs meeting GS > 0.2 and MM > 0.8 in WGCNA analysis were applied to univariate Cox regression. Then the genes with p value less than 0.2 in univariate Cox regression were considered as candidates, and inputted to multivariate Cox regression, which finally identified 15 genes (*FNDC1*, *FRMD6*, *FBN1*, *RAB31*, *GLT8D2*, *COL1A2*, *GLIS2*, *COL8A1*, *GPC6*, *COL3A1*, *PRICKLE1*, *FSTL1*, *HLX*, *IGFBP7*, and *EFS*) with a p value less than 0.05 in multivariate Cox regression. Next, the CAFGs scoring system was constructed based on the 15 genes and corresponding regression coefficients in multivariate Cox regression, as follows:

$${\text{CAFGs}} {\text{score}}=\sum_{i}\mathrm{Coefficient of} \left(i\right)\times \mathrm{Expression of gene} (i)$$

The regression coefficient of the gene was designated (*i*) in the multivariate Cox proportional hazards regression.

### Survival analysis

A total of 864 samples in the combined GEO cohort, 435 samples in the TCGA COAD cohort, have overall survival (OS) data (Supplementary Data 11). In addition, the recurrence-free survival (RFS) data in GSE39582; disease-free survival (DFS) data in GSE17536 and GSE17537; and disease-specific survival (DSS) data in GSE17536 are summarized in Supplementary Data 11, which were used to validate prognostic power of the CAFGs scoring system. The survival time was converted to months format, and patients with survival time less than 1 month were removed during survival analysis. Based on the optimal cutoff value identified by the *survminer* package, the patients were divided into high and low CAFGs score groups. Log-rank test was used to evaluate statistical differences. Kaplan–Meier (KM) plots were visualized using the *survminer* package.

### Inferring the consensus molecular subtypes (CMS) classification

The consensus molecular subtypes (CMS), a widely used classification system currently available for CRC, has strong prognostic implications in clinical application (Guinney et al. 2015). There are four subtypes of CMS, including CMS1 (MSI immune), CMS2 (canonical), CMS3 (metabolic), and CMS4 (mesenchymal). Among them, CMS4 is characterized by prominent transforming growth factor β (TGF-β) activation, stromal invasion, angiogenesis, poor OS, and RFS. In this study, we inferred CMS classification based on transcriptome data using R package *CMScaller* with the default parameter (Eide et al. 2017).

### ICB response prediction

A predictive algorithm known as the Tumor Immune Dysfunction and Exclusion (TIDE) algorithm was utilized to forecast the response to immune checkpoint blockade (ICB) by analyzing the gene expression profiles related to T-cell dysfunction (dysfunction) and exclusion (exclusion). A lower TIDE score indicates a more favorable immunotherapy response. The scores of T-cell dysfunction, T-cell exclusion, and TIDE were obtained from the TIDE website. The IMvigor210 cohort, a vast population of patients with metastatic urothelial cancer receiving anti-PD-L1 therapy (atezolizumab), was downloaded from the Creative Commons 3.0 license. GSE78220 is a cohort of pre-treatment melanomas receiving anti-PD-1 therapy.

### Statistical analysis

All analyses were performed in R 4.0.3. The Wilcox test was used to test the difference between two groups. The log-rank and Pearson test were used in KM survival and correlation analyses, respectively. In present study, heatmaps were visualized with the *ComplexHeatmap* package (Gu et al. 2016). The *ggplot2* package was used to visualize boxplots, scatter plots, and Sankey plots. *, **, ***, and **** represent a *p* value less than 0.05, 0.01, 0.001, and 0.0001, respectively.

## Supplementary Information

Below is the link to the electronic supplementary material.

Supplementary Figure 1: Processes of constructing CAF clusters. (A-D) Consensus matrixes of each k (k =2–5) in the combined GEO cohort. (E) Empirical cumulative distribution function plot displays consensus distributions for each k. When k=2, the distribution reaches an approximate maximum, indicating the cluster result is most stable. (TIF 1993 KB)

Supplementary Figure 2: Immune characterization between CAF clusters. (A) Heatmap shows the mRNA expressions of 122 immunomodulators between the CAF clusters. (B) The differences of enrichment scores of adaptive and innate immunity between CAF clusters inferred by ssGSEA analysis. (TIF 4049 KB)

Supplementary Figure 3: Processes of constructing CAFGs clusters. (A-D) Consensus matrixes of each k (k =2–5) in the combined GEO cohort. (E) Empirical cumulative distribution function plot displays consensus distributions for each k. When k=2, the distribution reaches an approximate maximum, indicating the cluster result is most stable. (TIF 1869 KB)

Supplementary Figure 4: Immune characterization between CAFGs clusters. (A) The heatmap reveals the differences of 11 critical biological pathways between CAFGs clusters. (B) The mRNA expression levels of several common inhibitory immune checkpoints between the CAFGs clusters. (TIF 3662 KB)

Supplementary Figure 5: Clinical significance of CAFGs scoring system in GSE39582. (A) The expression levels of CAFs markers between patients with high and low CAFGs scores in GSE39582. (B) The OS analysis of CAFGs scores in GSE39582. (C-D) KM plots shows the prognosis value of CAFGs scores in early and advanced stages in GSE39582. (E) The distribution of CAFGs scores in different groups of TNM stages, and CMS classification. (TIF 499 KB)

Supplementary Figure 6: Clinical significance of CAFGs scoring system in TCGA COAD. (A, B) The boxplot shows the CAFGs scores in different groups of M stage, and venous invasion. (TIF 531 KB)

Supplementary Figure 7: Immune characterization of CAFGs scoring system. (A) GSVA analysis shows the representative hallmark pathways that differs between high and low CAFGs scores groups. Hallmarks gene sets from the MsigDB databases were used. (B) The mRNA expressions of common ICI genes between high and low CAFGs scores groups. (TIF 2274 KB)

Supplementary Figure 8: Expression levels of 15 model genes across different cell clusters illustrated in UMAP plots. (TIFF 2569 KB)

Supplementary Data1: 596 differentially expressed genesbetween CAF and NF in CRC from a previous study. (csv 7 KB)

Supplementary Data2: 115 potential prognostic CAF genes used as the input genes of unsupervised clustering. (csv 2 KB)

Supplementary Data3: GO terms enriched in blue module. (csv 17 KB)

Supplementary Data4: KEGG terms enriched in blue module. (csv 7 KB)

Supplementary Data5: 43 genes meeting unicox p value less than 0.01 used as the input genes of unsupervised clustering. (csv 1 KB)

Supplementary Data6: The expression values of these CAFGs model genes and their corresponding regression coefficients. (csv 1 KB)

Supplementary Data7: High and low CAFGs score groups in multiple CRC cohorts. (xlsx 452 KB)

Supplementary Data8: CMS subtypes in multiple CRC cohorts. (xlsx 182 KB)

Supplementary Data9: 122 immunomodulators collected from previous studies. (xlsx 12 KB)

Supplementary Data10: Survival data of multiple CRC cohorts. (xlsx 73 KB)

## Data Availability

The original data presented in the study can be downloaded from GEO and TCGA websites.
