# Supplementary figures and images for: A comprehensive multi-omics analysis identifies a robust scoring system for cancer-associated fibroblasts and intervention targets in colorectal cancer
Source: J Cancer Res Clin Oncol. 2024 Mar 13;150(3):124. doi: 10.1007/s00432-023-05548-7 (PMC10937804; doi:10.1007/s00432-023-05548-7)

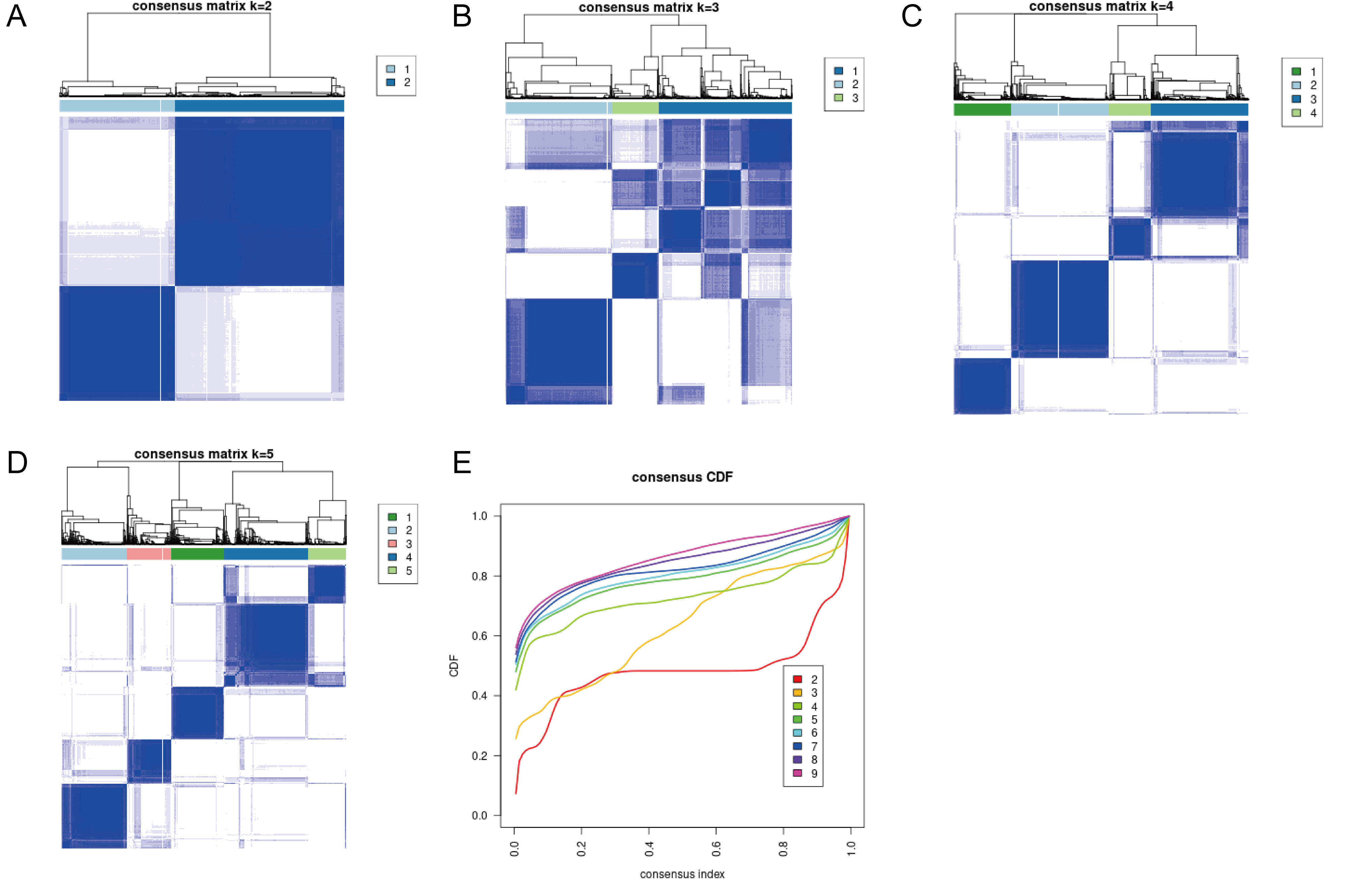

Supplement: Supplementary file 1 — Supplementary Figure 1: Processes of constructing CAF clusters. (A-D) Consensus matrixes of each k (k =2–5) in the combined GEO cohort. (E) Empirical cumulative distribution function plot displays consensus distributions for each k. When k=2, the distribution reaches an approximate maximum, indicating the cluster result is most stable. (TIF 1993 KB) [file 432_2023_5548_MOESM1_ESM.tif]

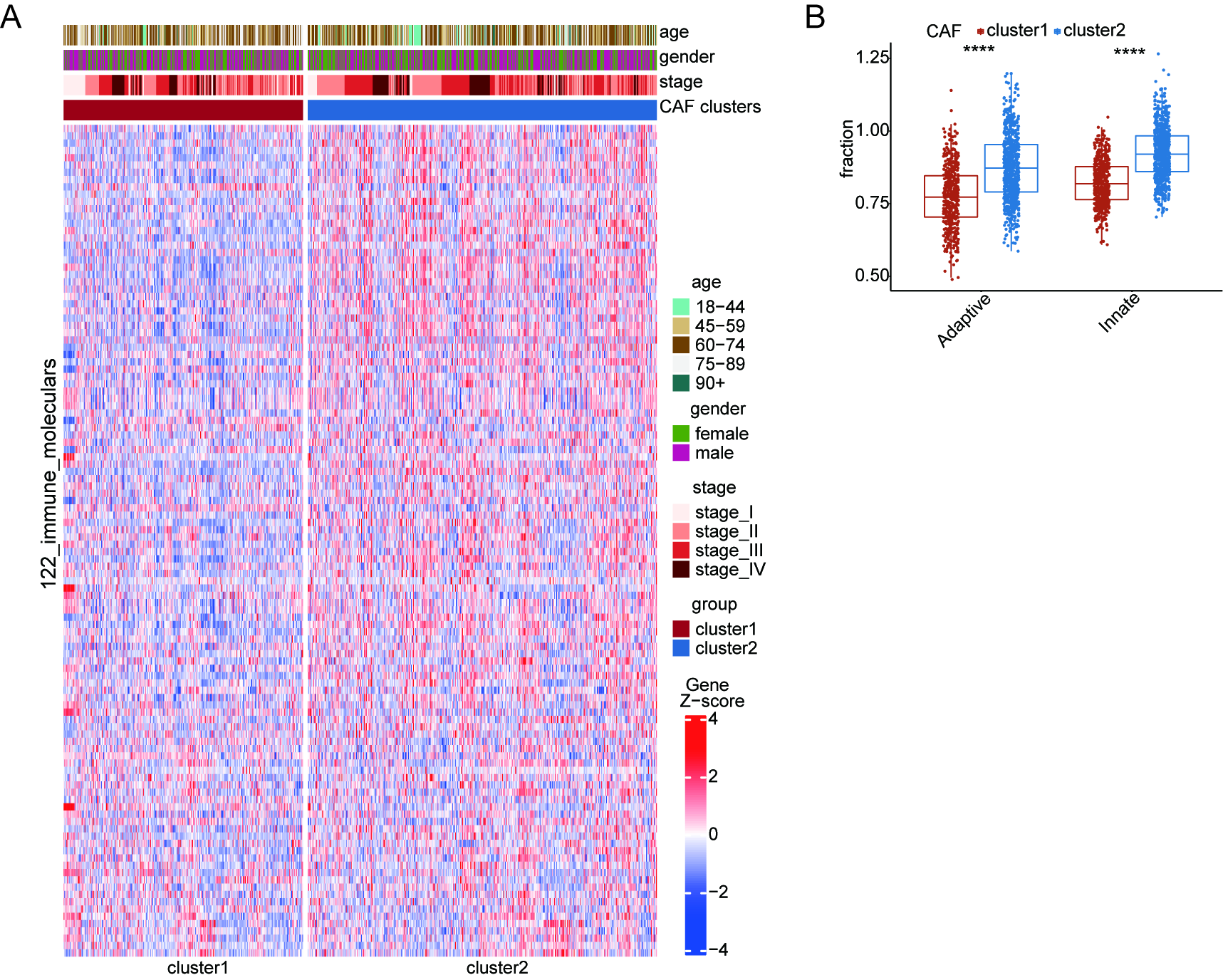

Supplement: Supplementary file 2 — Supplementary Figure 2: Immune characterization between CAF clusters. (A) Heatmap shows the mRNA expressions of 122 immunomodulators between the CAF clusters. (B) The differences of enrichment scores of adaptive and innate immunity between CAF clusters inferred by ssGSEA analysis. (TIF 4049 KB) [file 432_2023_5548_MOESM2_ESM.tif]

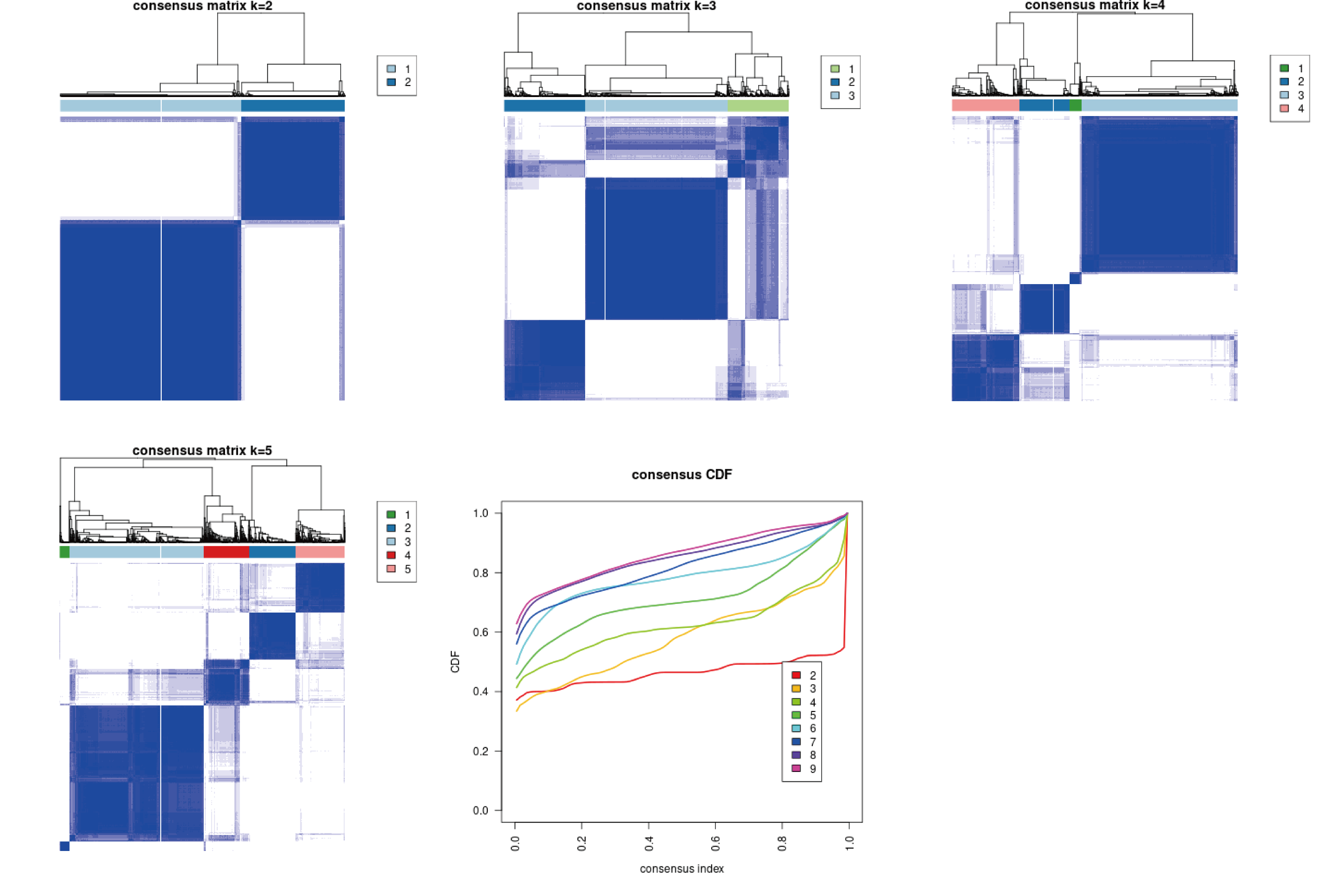

Supplement: Supplementary file 3 — Supplementary Figure 3: Processes of constructing CAFGs clusters. (A-D) Consensus matrixes of each k (k =2–5) in the combined GEO cohort. (E) Empirical cumulative distribution function plot displays consensus distributions for each k. When k=2, the distribution reaches an approximate maximum, indicating the cluster result is most stable. (TIF 1869 KB) [file 432_2023_5548_MOESM3_ESM.tif]

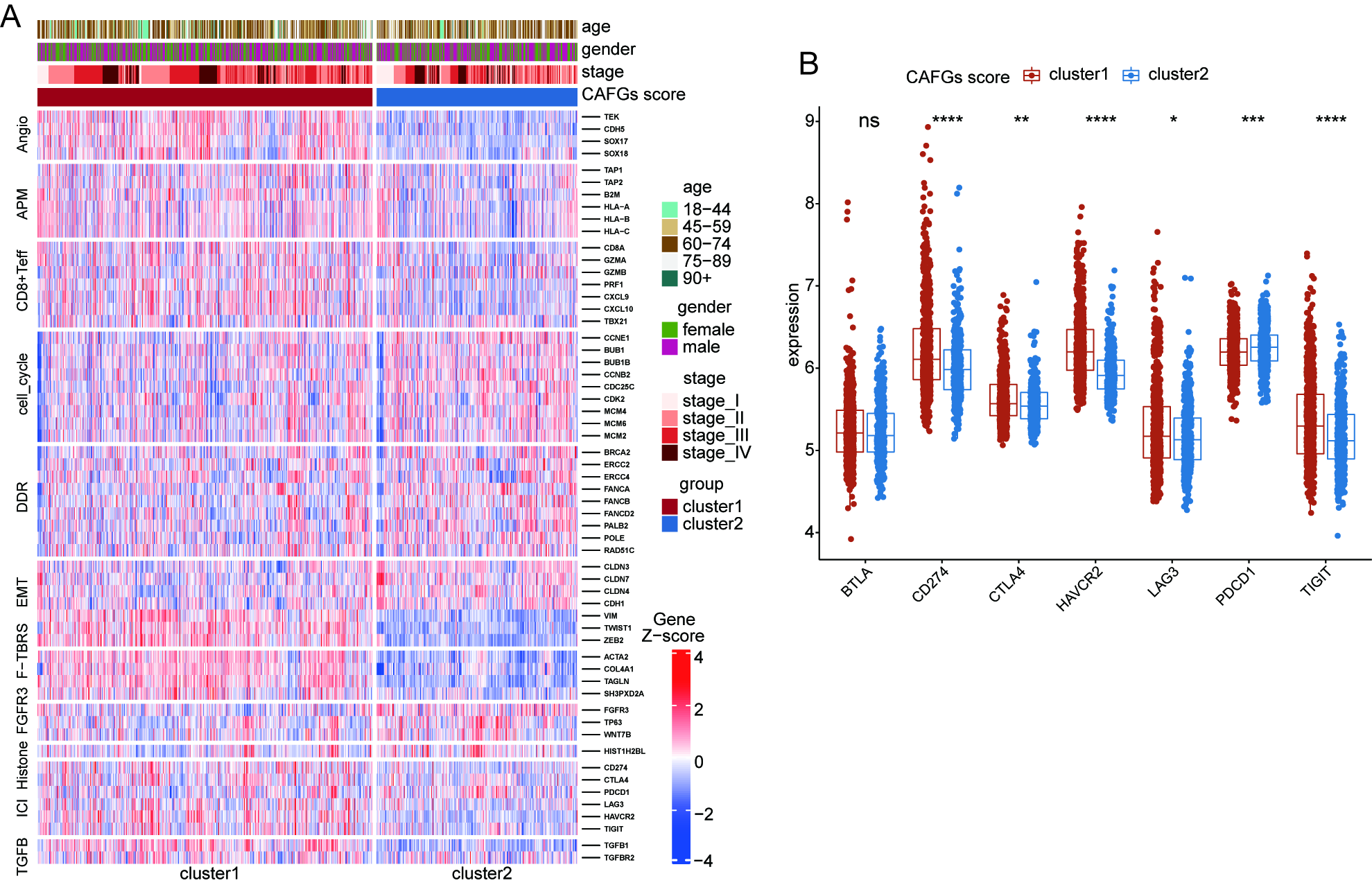

Supplement: Supplementary file 4 — Supplementary Figure 4: Immune characterization between CAFGs clusters. (A) The heatmap reveals the differences of 11 critical biological pathways between CAFGs clusters. (B) The mRNA expression levels of several common inhibitory immune checkpoints between the CAFGs clusters. (TIF 3662 KB) [file 432_2023_5548_MOESM4_ESM.tif]

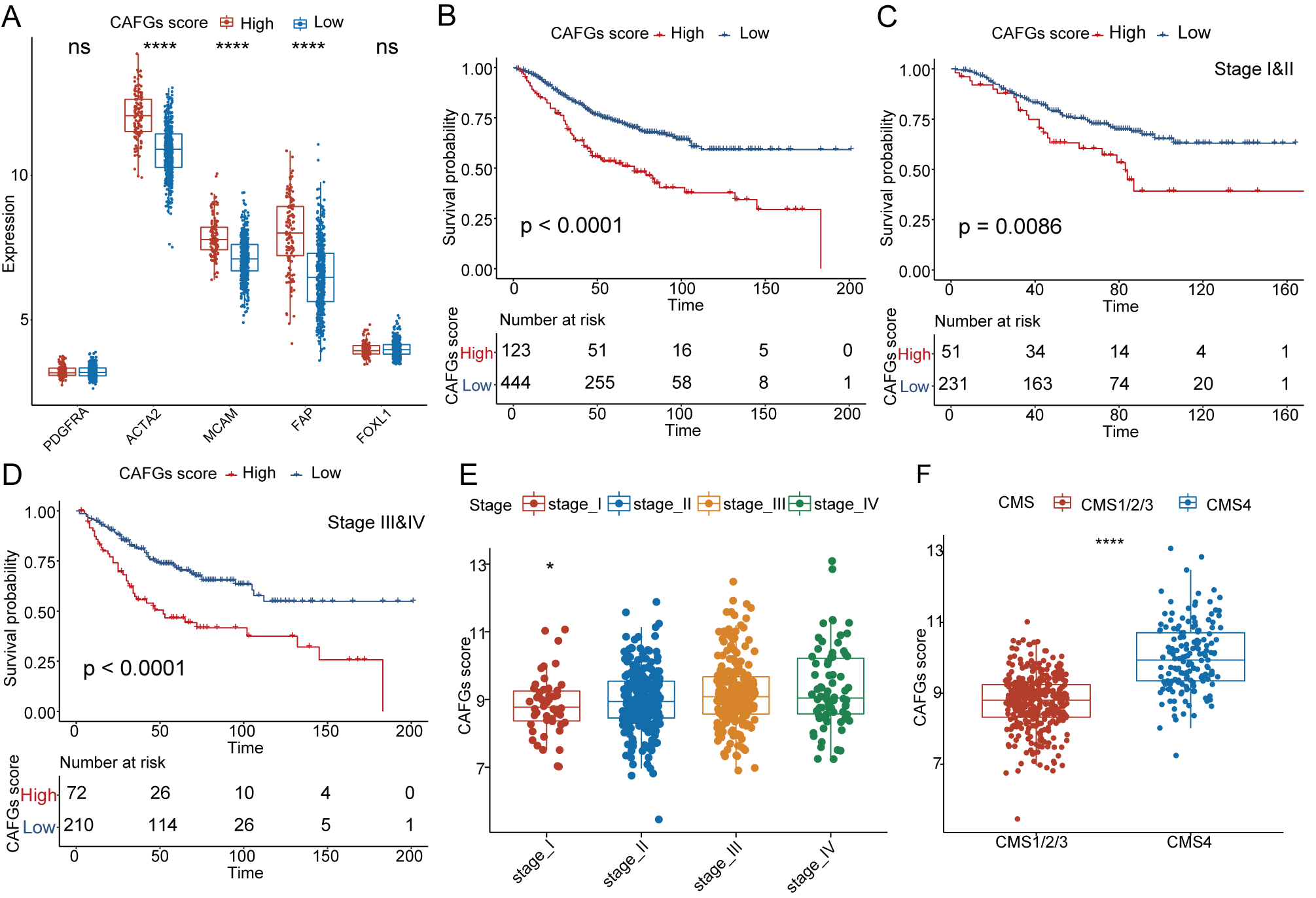

Supplement: Supplementary file 5 — Supplementary Figure 5: Clinical significance of CAFGs scoring system in GSE39582. (A) The expression levels of CAFs markers between patients with high and low CAFGs scores in GSE39582. (B) The OS analysis of CAFGs scores in GSE39582. (C-D) KM plots shows the prognosis value of CAFGs scores in early and advanced stages in GSE39582. (E) The distribution of CAFGs scores in different groups of TNM stages, and CMS classification. (TIF 499 KB) [file 432_2023_5548_MOESM5_ESM.tif]

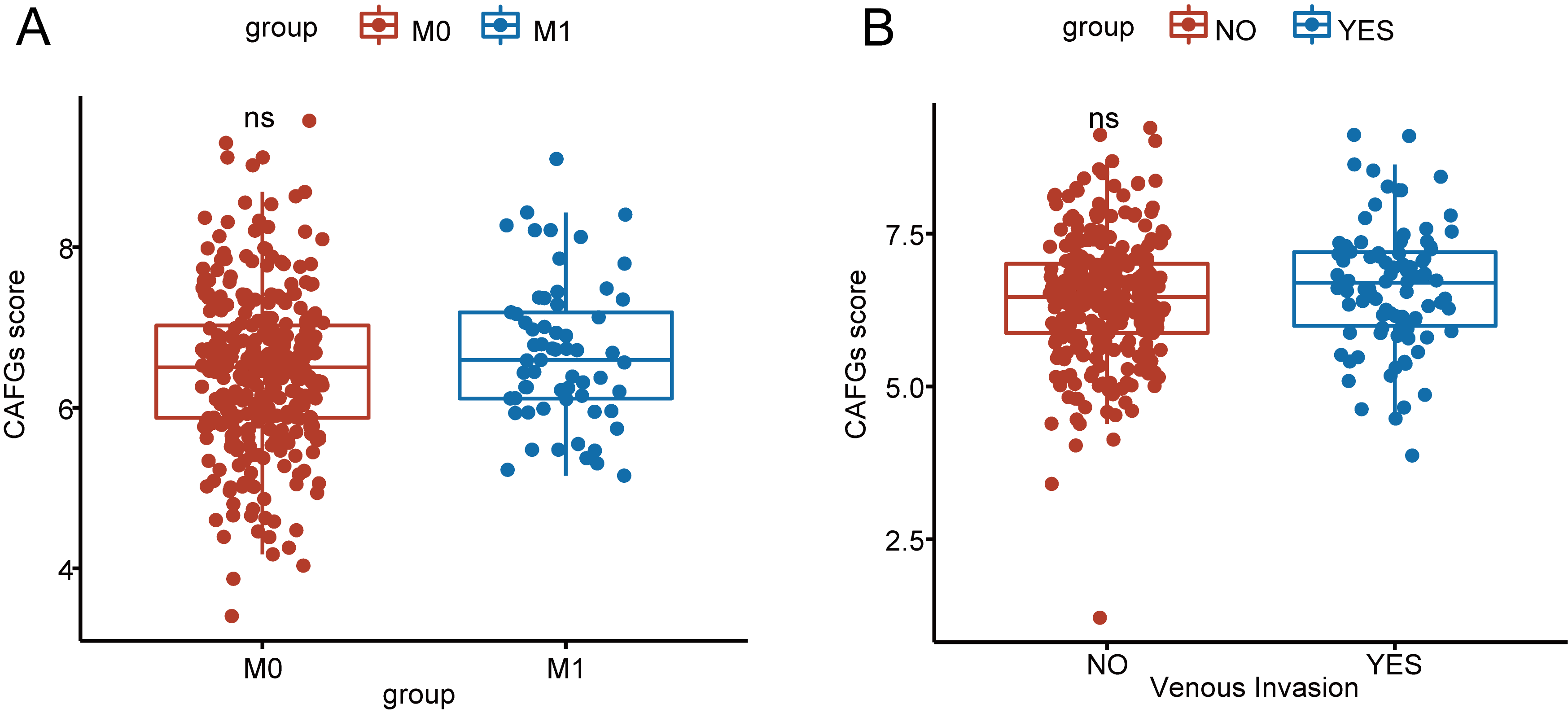

Supplement: Supplementary file 6 — Supplementary Figure 6: Clinical significance of CAFGs scoring system in TCGA COAD. (A, B) The boxplot shows the CAFGs scores in different groups of M stage, and venous invasion. (TIF 531 KB) [file 432_2023_5548_MOESM6_ESM.tif]

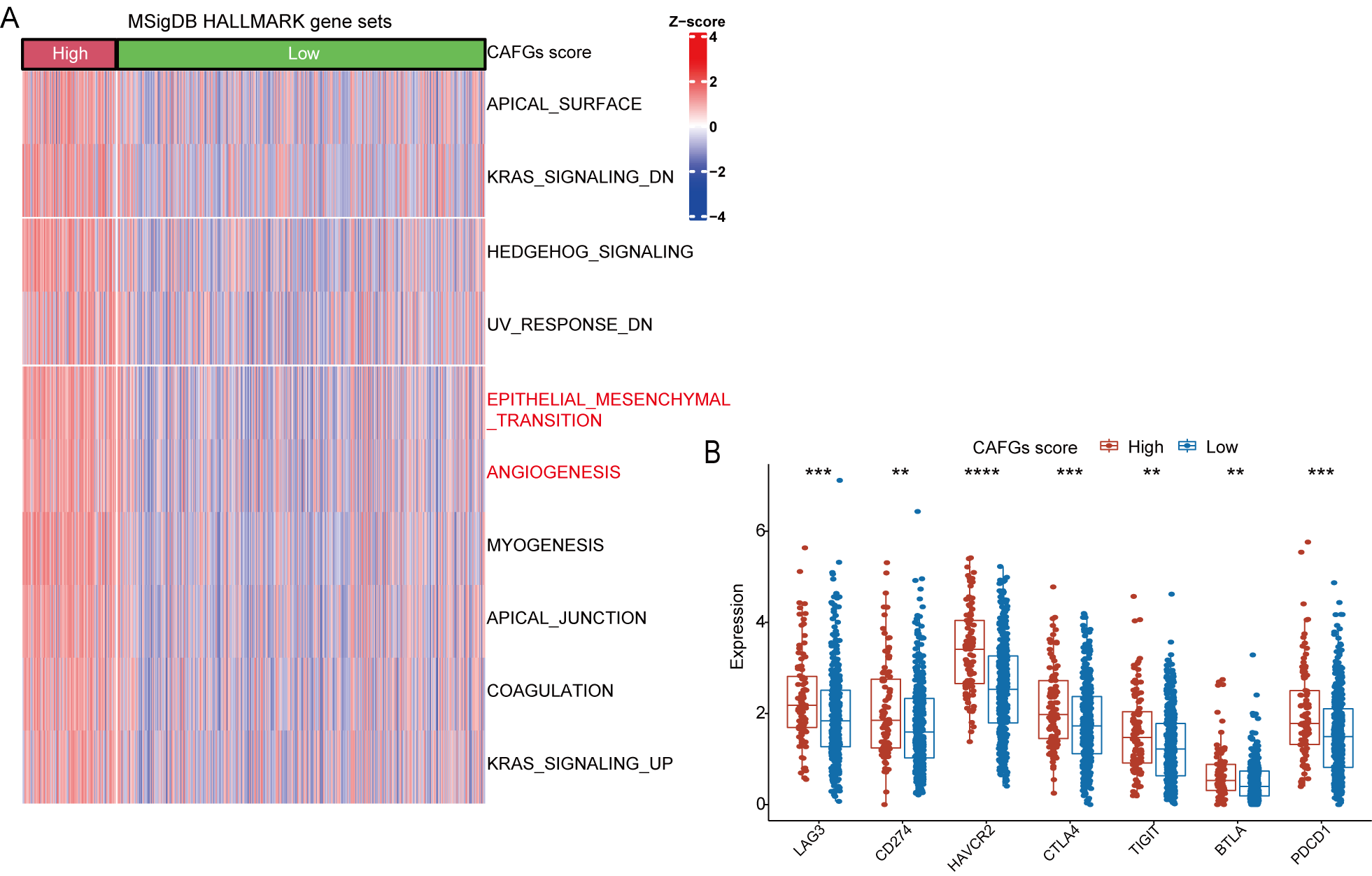

Supplement: Supplementary file 7 — Supplementary Figure 7: Immune characterization of CAFGs scoring system. (A) GSVA analysis shows the representative hallmark pathways that differs between high and low CAFGs scores groups. Hallmarks gene sets from the MsigDB databases were used. (B) The mRNA expressions of common ICI genes between high and low CAFGs scores groups. (TIF 2274 KB) [file 432_2023_5548_MOESM7_ESM.tif]

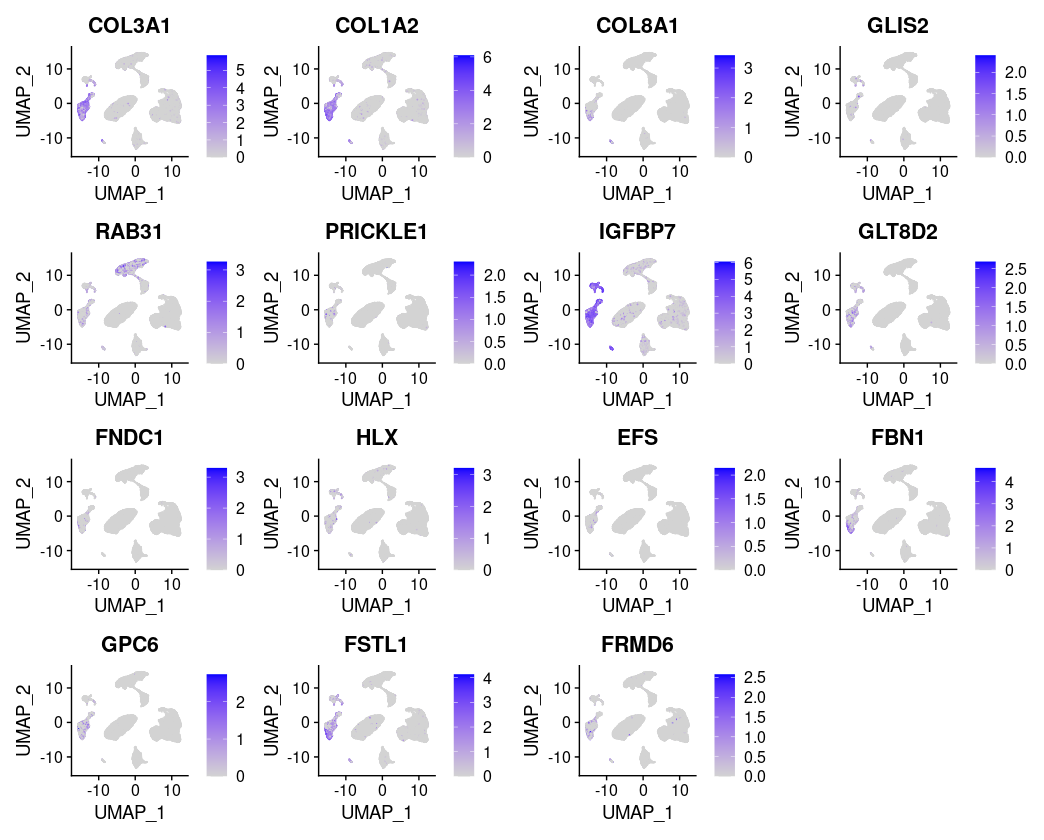

Supplement: Supplementary file 8 — Supplementary Figure 8: Expression levels of 15 model genes across different cell clusters illustrated in UMAP plots. (TIFF 2569 KB) [file 432_2023_5548_MOESM8_ESM.tiff]
